# Digital twinning of cardiac electrophysiology for congenital heart disease

**DOI:** 10.1101/2023.11.27.568942

**Published:** 2023-11-28

**Authors:** Matteo Salvador, Fanwei Kong, Mathias Peirlinck, David W. Parker, Henry Chubb, Anne M. Dubin, Alison Lesley Marsden

**Affiliations:** 1 Department of Bioengineering, Stanford University, California, USA; 2 Institute for Computational and Mathematical Engineering, Stanford University, California, USA; 3 Cardiovascular Institute, Stanford University, California, USA; 4 Pediatric Cardiology, Stanford University, California, USA; 5 Department of Biomechanical Engineering, Delft University of Technology, Delft, Netherlands; 6 Stanford Research Computing Center, Stanford University, California, USA

**Keywords:** Single Ventricle Physiology, Numerical Simulations, Neural Maps, Parameter Estimation, Uncertainty Quantification

## Abstract

In recent years, blending mechanistic knowledge with machine learning has had a major impact in digital healthcare. In this work, we introduce a computational pipeline to build certified digital replicas of cardiac electrophysiology in pediatric patients with congenital heart disease. We construct the patient-specific geometry by means of semi-automatic segmentation and meshing tools. We generate a dataset of electrophysiology simulations covering cell-to-organ level model parameters and utilizing rigorous mathematical models based on differential equations. We previously proposed Branched Latent Neural Maps (BLNMs) as an accurate and efficient means to recapitulate complex physical processes in a neural network. Here, we employ BLNMs to encode the parametrized temporal dynamics of in silico 12-lead electrocardiograms (ECGs). BLNMs act as a geometry-specific surrogate model of cardiac function for fast and robust parameter estimation to match clinical ECGs in pediatric patients. Identifiability and trustworthiness of calibrated model parameters are assessed by sensitivity analysis and uncertainty quantification.

## 1 Introduction

The combination of physics-based and statistical modeling in cardiovascular medicine has the potential to shape the future of cardiology [9]. In this framework, a synergistic use of multiphysics and multiscale mathematical models for cardiac function [13, 18, 39, 41, 57] and machine learning-based methods, such as Gaussian processes emulators [30, 37, 59] and Neural Networks (NNs) [35, 47], enables the design of efficient computational tools that are compatible with the computer resources and time frames required in clinical applications. In the foreseeable future, a continuous, bi-directional interaction between patient-specific data and Artificial Intelligence-enriched computer models incorporating biophysically detailed and anatomically accurate knowledge would enable the vision of precision medicine [34, 38, 44]. Personalized treatment and surgical planning may be delivered by leveraging different mathematical methods, such as sensitivity analysis, parameter inference and uncertainty quantification [19, 25, 49, 54].

Several mathematical tools have been proposed to better understand and treat different groups of adult cardiac pathologies [34]. Electrophysiology simulations play an important role in the assessment of rhythm disorders. They are used for cardiac resynchronization therapy [58], arrhythmia risk stratification [2, 50], and definition of optimal ablation strategies [43]. Nevertheless, in silico numerical simulations and treatment modalities in pediatrics and congenital heart disease are less common or even not established [8, 24, 62].

Congenital heart defects (CHDs) are the most common birth defects and are characterized by cardiac anatomical abnormalities that can severely impact cardiac function [29]. Patients with CHDs often have a unique and peculiar combination of cardiac defects that warrant personalized treatment planning in clinically-relevant time frames. Digital twinning of cardiac function thus holds particular promise for these patients [62].

In this work we introduce a novel digital twin of a pediatric patient with hypoplastic left heart syndrome (HLHS), a complex form of CHD where the left ventricle of the patient is severely underdeveloped, leading to a number of morbidities and elevated mortality risk. Our pipeline seamlessly combines:

- Semi-automatic segmentation and mesh generation tools suited for pediatric patients with CHD [27]
- A physiologically-based mathematical formulation of cardiac electrophysiology deriving from the monodomain equation [44, 51] coupled with the ten Tusscher-Panfilov [63] ionic model
- A recently proposed scientific machine learning method, namely Branched Latent Neural Maps (BLNMs) [53], to build an accurate and efficient dynamic surrogate model of cardiac function
- Shapley effects [56] and Hamiltonian Monte Carlo (HMC) [5, 21] to perform patient-specific sensitivity analysis, fast and robust parameter estimation with uncertainty quantification while matching clinical 12-lead electrocardiograms (ECGs)

This digital twin can be employed to simulate different scenarios of clinical interest in silico, as HLHS patients may experience different forms of electrophysiological comorbidities [14], including ventricular arrhythmias and dyssynchrony [62]. Therefore, personalized electrophysiology simulations may provide virtual pre- and post-operative guidance in this understudied patient cohort [32].

## 2 Results

**Figure 1:**
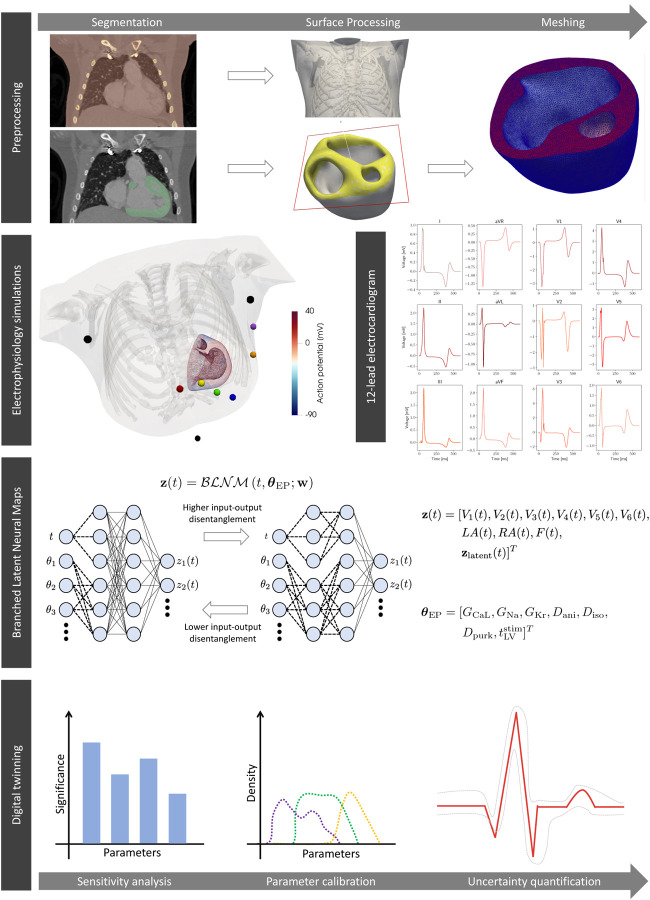
Sketch of the computational pipeline. We reconstruct the patient-specific geometry with HLHS from imaging. We generate a dataset of electrophysiology simulations encompassing cell-to-organ variability in model parameters. We train a BLNM that effectively reproduces 12-lead ECGs while covering model variability. We employ the BLNM for digital twinning.

In Figure 1 we depict our computational pipeline to build digital twins of cardiac electrophysiology for congenital heart disease patients. This pipeline covers all the relevant aspects of digital twinning: image segmentation and mesh generation, mathematical and numerical physics-based modeling, surrogate model training, sensitivity analysis and robust parameter calibration with uncertainty quantification.

### 2.1 Pre-processing

Figure 1 (first row) shows the heart-torso model of a 7-year-old female pediatric patient with HLHS constructed from the computerized tomography (CT) scan of the patient using our semi-automatic model construction pipeline [27].

### 2.2 Cardiac electrophysiology modeling

We run 200 numerical simulations on the patient-specific heart-torso geometry (see Figure 1, second row), spanning seven relevant electrophysiology parameters of the physics-based model at the microscopic scale and organ level. We collect the corresponding in silico 12-lead ECGs. In Table 1 we report descriptions, ranges, and units for the seven model parameters that we explore via latin hypercube sampling for the dataset generation.

**Figure 2:**
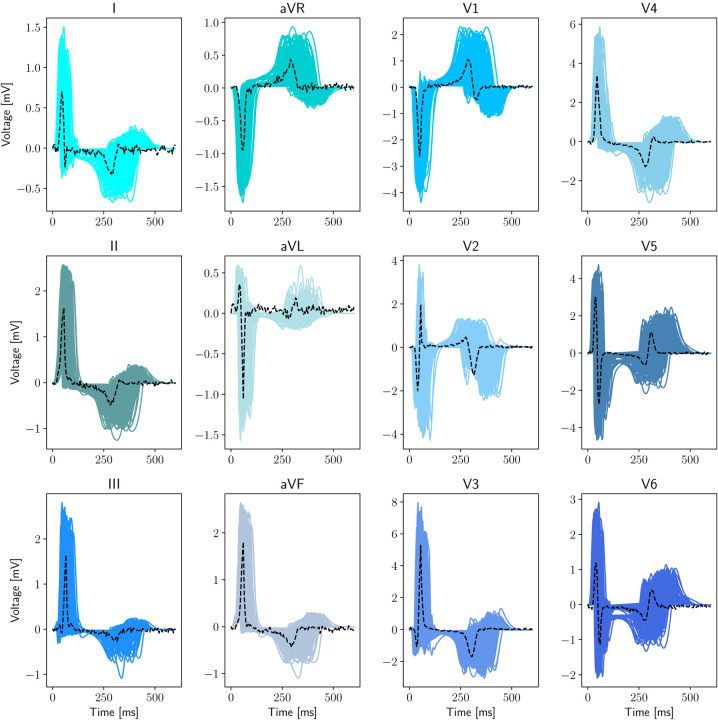
Physics-based electrophysiological modeling dataset generation. Full dataset containing 200 in silico precordial and limb leads recordings (blue, solid) and patient-specific 12-lead ECGs (black, dashed).

In Figure 2 we depict the ensemble of the resulting in silico 12-lead ECGs together with the clinical recordings. We point out that the patient-specific 12-lead ECGs are contained within the pseudopotentials variability spanned by the electrophysiology simulations, manifesting various morphologies in the QRS complex, that is ventricular depolarization, and T wave, that is ventricular repolarization. The patient diagnosis reports rhythm disorders, atrial enlargement, left and right ventricular hypertrophy, along with severe abnormalities in the ECGs. Specifically, there are signs of prolonged PR interval, ST segment depression and T wave inversion.

**Figure 3:**
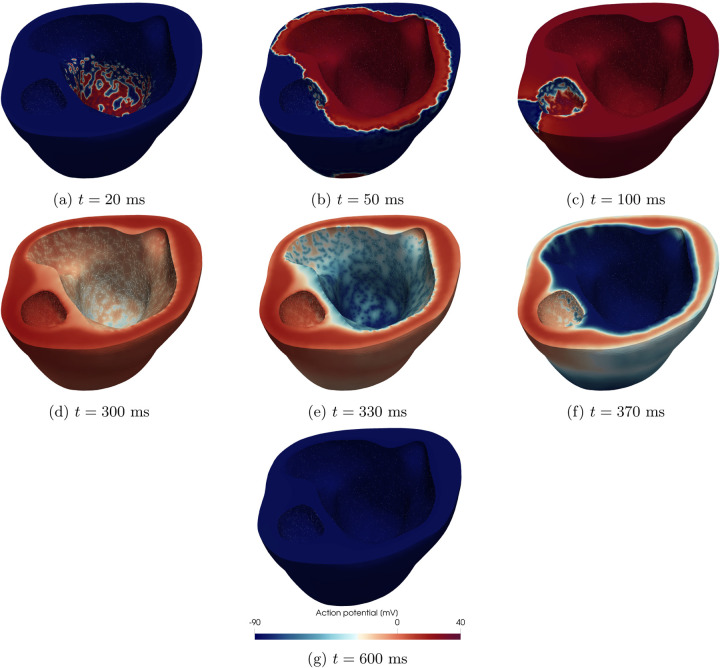
Physics-based electrophysiological modeling. Spatio-temporal membrane action potential evolution for one electrophysiology simulation in the dataset performed on the HLHS pediatric patient.

In Figure 3 we show the simulated spatio-temporal transmembrane potential evolution on the patient-specific pediatric model for a single instance of model parameters. Specifically, we always simulate the sinus rhythm behavior over a cardiac cycle. Figure 3 focuses on the ventricular depolarization phase, where the electric signal propagates from the 1D Purkinje network at the two endocardia towards the myocardium, as well as the ventricular repolarization phase, when the transmembrane potential comes back to its resting state (i.e. approximately −90 mV).

### 2.3 Branched Latent Neural Maps

We train BLNMs, which are represented by feedforward partially-connected NNs, to encode the temporal dynamics of the 12-lead pseudo-ECGs computed with the physics-based mathematical model while also covering model variability from the cellular to the tissue level (see Figure 1, third row). Once trained, BLNMs act as a surrogate model for cardiac electrophysiology function that can be queried on new parameter instances to provide faster than real-time in silico 12-lead ECGs.

In order to identify the optimal set of BLNM hyperparameters, which are the number of layers, number of neurons, number of states, and disentanglement level in the NN structure, we employ a K-fold (K=5) cross validation over 150 multiscale physics-based electrophysiology simulations. The hyperparameter search space is given by a four-dimensional hypercube, where we run 50 instances of Latin Hypercube Sampling and we pick the BLNM configuration providing the lowest generalization error. For each configuration of hyperparameters, we sample the dataset with a fixed time step of Δt=5ms and we perform 10,000 iterations of the second-order Broyden-Fletcher-Goldfarb-Shanno (BFGS) optimizer. In Table 2 we report the initial hyperparameters ranges for tuning and the final optimized values.

Then, we train a final optimized BLNM encompassing the whole dataset of 150 multiscale physics-based electrophysiology simulations using 50,000 BFGS iterations. The non-dimensional Mean Square Error (MSE) on a testing set comprised of 50 additional numerical simulations unseen during the training stage, and Latin Hypercube sampled from the same parameter space in Table 1, is equal to $5\cdot 10^{-4}$.

### 2.4 Parameter estimation

We employ the optimized BLNM to find an initial guess for the seven model parameters that results in a computational pseudo-ECG that best matches the clinically observed 12-lead ECG dynamics of the CHD patient. The estimated model parameters are reported in Table 3.

Even though the relative heart orientation and lead placements significantly influence ECGs [66], and as such may require additional parameters to calibrate, the information retrieved from the CT scan and patient diagnosis allow us to determine these quantities with a small degree of uncertainty. This motivates our focus in the estimation process on the cell-to-organ level electrophysiology model parameters which are assessed as important in previous studies [10,60].

### 2.5 Sensitivity analysis

**Figure 4:**
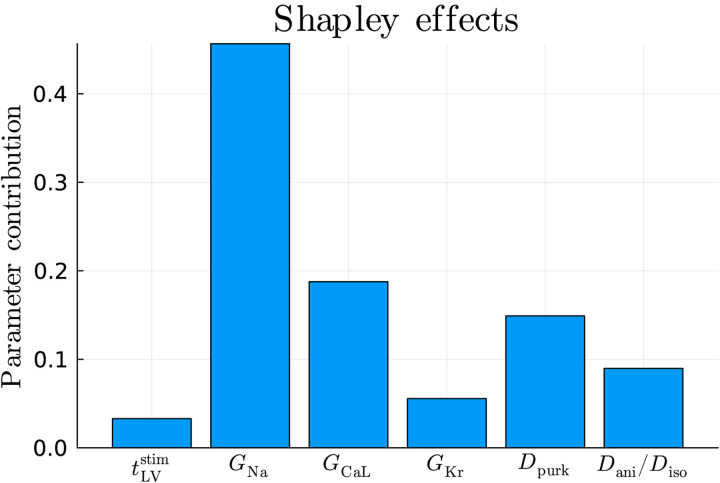
Sensitivity analysis for the seven model parameters encoded in the BLNM via Shapley effects.

Starting from the parameter calibration shown in Table 3, we compute Shapley effects for each model parameter for cardiac electrophysiology, assuming independence among them as they act in different terms and equations of the physics-based mathematical model (see Figure 1, third row). In Figure 4 we show how each parameter contributes in matching electrophysiology simulations with the clinical 12-lead ECGs, i.e. in the minimization of the MSE between BLNM outputs and our patient-specific observations. The sodium current conductance $G_{Na}$ plays a dominant role, followed by the L-type calcium ion channel conductance $G_{CaL}$ and the different conductivities $D_{\text{ani}},D_{\text{iso}}$ and $D_{\text{purk}}$. Noteworthy, the interventricular activation dyssynchrony $t_{LV}^{\text{stim}}$ plays a minor role in the calibration process. This is motivated by the dimension of the right ventricle, which mostly dictates the activation sequence with respect to the small (underdeveloped) left ventricle.

### 2.6 Uncertainty quantification

**Figure 5:**
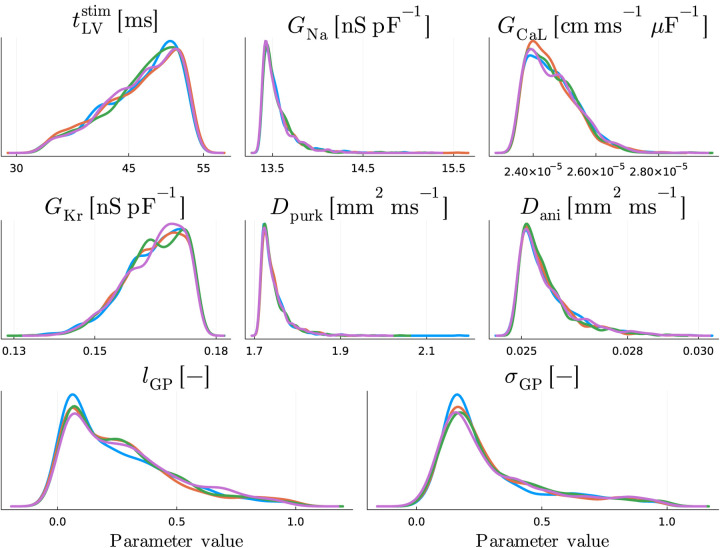
Inverse uncertainty quantification: parameter uncertainty. One-dimensional views of the posterior distribution. Different colors represent different HMC chains.

**Figure 6:**
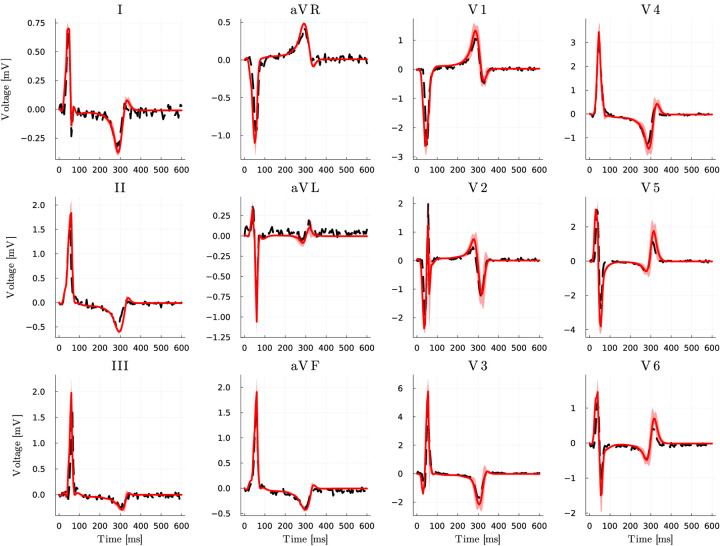
Inverse uncertainty quantification: matching clinical data. Clinical recordings (dashed, black) and mean estimation (red, solid) of 12-lead ECGs for the HLHS pediatric patient via HMC. Light red encompasses the variability between mean minus/plus five standard deviations.

In Figures 5 and 6 we show the results of our inverse uncertainty quantification, where we quantify how uncertainty in matching 12-lead ECGs propagates to uncertainty in the estimated model parameters. We account for both BLNM surrogate modeling error, via Gaussian processes (GPs), and measurement error in the clinical recordings.

From Figure 5, we see that the posterior distributions of all model parameters $\theta_{EP}$, along with the correlation length $l_{GP}$ and amplitude $\sigma_{GP}$, converged well towards highly similar unimodal distributions across all chains. The average value of $\sigma_{GP}^{2}$ is approximately equal to 0.08, which is two orders of magnitude higher than the BLNM testing error $\left(5\cdot 10^{-4}\right)$, as the GP encodes the maximum BLNM uncertainty from each single lead individually and by also considering all possible correlations among the 12 leads, given the full covariance matrix in the multivariate normal distribution (see Section 3.6).

In Figure 6 we depict the clinical vs. in silico 12-lead ECGs, generated with the BLNM over the posterior distribution of model parameters. We see that the numerical simulations are in good agreement with the patient-specific recordings and show small variability between the five standard deviations from the average value.

### 2.7 In silico clinical trial

**Figure 7:**
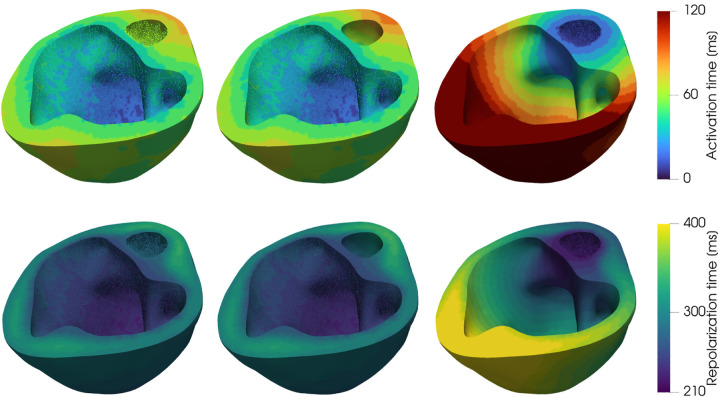
Running an in silico clinical trial. Activation (top) and repolarization (bottom) maps with personalized model parameters (left), left bundle branch block (center) and right bundle branch block (right).

In Figure 7 we show the results of three numerical simulations in which we analyze different scenarios of clinical interest pre-operatively on the HLHS pediatric patient. Specifically, we depict activation and repolarization times for the electrophysiology simulation with the calibrated model parameters $\theta_{EP}$ and by inducing either a left or a right bundle branch block, where the left (respectively, right) Purkinje fascicles are inhibited. We notice that, for this patient-specific case, the role of the Purkinje network in the left ventricle is very limited and that the activation sequence is highly similar with and without a full left bundle branch block.

### 2.8 Computational costs

In Table 4 we detail the computational costs and resources required by each step of the digital twinning process. The most expensive part resides in the physics-based computational electrophysiology modeling dataset generation, which makes use of high-performance computing given the stiffness and complexity of the underlying mathematical model. On the other hand, training a BLNM and employing it for robust Bayesian parameter estimation and sensitivity analysis are more tractable tasks that can be carried out within a few hours or minutes on a local machine. Using the physics-based mathematical model throughout parameter calibration with uncertainty quantification and sensitivity analysis would be computationally intractable and unaffordable given the extensive number of queries that Shapley effects and HMC require to show robustness and convergence in the provided results (see Methods section).

### 3 Methods

#### 3.1 Pre-processing

We reconstruct the heart-torso model from the CT scan of a 7-year-old female pediatric patient with HLHS in a semi-automatic manner [27]. Namely, we train a NN based on the classic UNet architecture [23] to automatically segment the myocardium from CT images. The UNet is trained using a publicly available dataset [67] that provided CT images and ground truth segmentation for 110 patients with age between 1 month and 40 years, combined with our private HLHS dataset containing the images and segmentation of 5 patients. Given the intrinsic segmentation challenges of cardiac structures in both young and CHD patients [40], we subsequently examine and improve the UNet-produced segmentations to more closely match with the CT scan. We automatically extract the surface meshes from the segmentations using the marching cube algorithm [31] and truncate the base myocardium above a manually identified plane to create a biventricular surface mesh. We subsequently use TetGen [55] to create the tetrahedral volumetric mesh with a maximum edge size of 1 mm [53, 62]. The torso model is created semi-automatically from the images using threshold-and region-growing-based segmentation methods. Images and associated clinical data were obtained under an IRB-approved protocol at Stanford University.

### 3.2 Cardiac electrophysiology modeling

We detail the physics-based mathematical model, along with its numerical discretization, that is employed to perform electrophysiology simulations on the HLHS pediatric patient.

### 3.2.1 Mathematical model

We consider the monodomain equation [44] coupled with the ten Tusscher-Panfilov ionic model [63] to describe the electric behavior in the heart-Purkinje system. This system of differential equations reads:

(1) $$\{\begin{array}{ll} \frac{\partial \Phi }{\partial t}+{ℐ}_{\text{ion}}\left(\Phi ,w,z\right)-\nabla \cdot \left(D_{\text{M}}\nabla \Phi \right)={ℐ}_{\text{app}}\left(x,t\right) & \text{ in Ω}\times \left(0,T\right],\\ \left(D_{\text{M}}\nabla \Phi \right)\cdot n=0 & \text{ on }\partial \Omega \times \left(0,T\right],\\ \frac{dw}{dt}=H\left(\Phi ,w,z\right) & \text{ in Ω}\times \left(0,T\right],\\ \frac{dz}{dt}=G\left(\Phi ,w,z\right) & \text{ in Ω}\times \left(0,T\right],\\ \Phi \left(x,0\right)=\Phi_{0}\left(x\right),w\left(x,0\right)=w_{0}\left(x\right),z\left(x,0\right)=z_{0}\left(x\right) & \text{ in Ω}. \end{array}$$

We always simulate a single heartbeat by fixing a final time $\;=T_{HB}=\;600\;ms$. The computational domain $\Omega \;=\Omega_{\text{purk}}\cup \Omega_{\text{myo}}$ is represented by the one-way coupled 1D Purkinje network and 3D biventricular patient-specific geometry.

Transmembrane potential Φ defines the electric signal at the Purkinje and myocardial level. The ten Tusscher-Panfilov ionic model is endowed with 18 variables, which are split in two different subsets. First, there is a vector $w=\left(w_{1},\ldots ,w_{M}\right)\;(M=12)$ of ion channel gating variables, which are probability density functions representing the fraction of open channels across the membrane of a single cardiac cell. Then, there is a vector $z=\left(z_{1},\ldots ,z_{P}\right)\;(P=6)$ of concentration variables representing relevant ionic species, such as sodium ${Na}^{+}$, intracellular calcium ${Ca}^{2+}$ and potassium $K^{+}$, which all play a major role in the metabolic processes [4], dictating heart rhythmicity or sarcomere contractility, and are generally targeted by pharmaceutical therapies. The specific mathematical formulation of the ten Tusscher-Panfilov ionic model defines the ordinary differential equations for H(Φ,w,z) and G(Φ,w,z), which describe the dynamics of gating variables and ionic concentrations respectively, along with the ionic current ${ℐ}_{\text{ion}}(\Phi ,w,z)$ [63]. An external applied current ${ℐ}_{\text{app}}(x,t)$ fires the electric signal in the Purkinje fibers.

The diffusion tensor is expressed as $D_{M}=D_{\text{iso}}I+D_{\text{ani}}f_{0}⊗f_{0}$ in $\Omega_{\text{myo}}$ and $D_{M}=D_{\text{purk}}I$ in $\Omega_{\text{purk}}$, where $f_{0}$ introduces the biventricular fiber field [42, 51]. $D_{\text{ani}},D_{\text{iso}},D_{\text{purk}}\in R^{+}$ dictate the anisotropic, isotropic and Purkinje conductivities, respectively.

The homogeneous Neumann boundary conditions prescribed at ∂Ω define the condition of an electrically isolated domain, where n is the outward unit normal vector to the boundary.

Following [51], the extracellular potential $\Phi_{e}$ defining the ECGs is computed in each lead location $x_{e}$ as:

(2) $$\Phi_{\text{e}}\left(x_{\text{e}}\right)=-\underset{\Omega }{\int }\nabla \Phi \cdot \nabla \frac{1}{∥x-x_{\text{e}}∥}dV,$$

where $e=\left\{V_{1},V_{2},V_{3},V_{4},V_{5},V_{6}\right\}$ and e={LA,RA,F} define six precordial leads and three limb leads located on the pediatric patient-specific torso model, shown in colored and black dots in Figure 1 (second row), respectively. From these lead locations, we computationally reconstruct three bipolar limb leads as:

(3) I=LA−RAII=F−RAIII=F−LA,

and three augmented limb leads as:

(4) aVL=(I−III)/2aVR=−(I+II)/2aVF=(II+III)/2.

The resulting set $ECG=\left\{V_{1},V_{2},V_{3},V_{4},V_{5},V_{6},I,II,III,aVL,aVR,aVF\right\}$ of computational pseudopotentials defines a comprehensive 12-lead ECG representation of the electrical activity in the patient-specific heart.

#### 3.2.2 Numerical discretization

We employ linear Finite Elements to discretize the spatial domain Ω in Equation (3.2.2). The tetrahedral tessellation defining the biventricular mesh has 933,916 cells and 158,277 DOFs, with a maximum mesh size of h=1mm. The left and right Purkinje bundles within the ventricular endocardia are generated by employing the fractal tree and projection algorithm proposed in [48], starting from the atrioventricular node. These left and right bundles are endowed with 14,820 elements (14,821 DOFs) and 67,456 elements (67,457 DOFs), respectively. Given the space resolution of the biventricular mesh, we apply non-Gaussian quadrature rules to recover convergent conduction velocities [62]. We consider a transmural variation of ionic conductances to differentiate epicardial, mid-myocardial and endocardial properties [63]. To solve Eq., we leverage an Implicit-Explicit time discretization scheme, where we first update the variables of the ionic model and then the transmembrane potential [13]. Specifically, in the monodomain equation, the diffusion term is treated implicitly and the ionic term is treated explicitly. The latter is discretized by means of the Ionic Current Interpolation scheme [28]. We prescribe the fiber distribution according to a Laplace-Dirichlet Rule-Based algorithm with $\alpha_{epi}=-60^{{}^{\circ}},\alpha_{\text{endo}}=60^{{}^{\circ}},\beta_{\text{epi}}=20^{{}^{\circ}}$ and $\beta_{\text{endo}}=-20^{{}^{\circ}}$ [42].

### 3.3 Branched Latent Neural Maps

We construct a geometry-specific surrogate model of cardiac function by building a feedforward partially-connected NN that explores the variability of our physics-based electrophysiology model detailed in Section 3.2 while structurally separating the role of temporal t and functional $\theta_{EP}$ parameters. This recent scientific machine learning tool, proposed in [53], allows for different levels of disentanglement between inputs and outputs. The surrogate model reads:

(5) $$z\left(t\right)=ℬℒ𝒩ℳ\left(t,\theta_{\text{EP}};w\right)\text{ for }t\in \left[0,T\right].$$

Weights and biases $w\in R^{N_{w}}$ encode the algebraic structures of a feedforward partially-connected NN, which represents a map $ℬℒ𝒩ℳ:R^{1+N_{𝒫}}\to R^{N_{z}}$ from time t and $N_{𝒫}=7$ cell-to-organ scale electrophysiology parameters $\theta_{EP}={\left[G_{CaL},G_{Na},G_{Kr},D_{\text{ani}},D_{\text{iso}},D_{\text{purk}},t_{LV}^{\text{stim}}\right]}^{T}\in \Theta \subset R^{N_{𝒫}}$ to an output vector $z(t)={\left[z_{\text{leads}}(t),z_{\text{latent}}(t)\right]}^{T}\in R^{N_{z}}$. This vector contains in silico precordial and limb leads recordings $z_{\text{leads}}(t)={\left[V_{1}(t),V_{2}(t),V_{3}(t),V_{4}(t),V_{5}(t),V_{6}(t),LA(t),RA(t),F(t)\right]}^{T}\in R^{9}$, where we use the original $LA\left(t\right),\;RA(t)$ and F(t) limb leads in place of the bipolar and augmented limb leads in order to reduce the dimensionality of the output. Indeed, we reconstruct $I\left(t\right),\;II\left(t\right),\;III\left(t\right),\;aVL\left(t\right),\;aVR(t)$ and aVF(t) a posteriori by means of Equations (3) and (4). Furthermore, vector z(t) leverages some $z_{\text{latent}}(t)$ latent variables that enhance the learned temporal dynamics by acting in regions with steep gradients [53].

We perform nonlinear optimization with the BFGS algorithm to tune NN parameters. In particular, we monitor the MSE of surrogate vs. physics-based ECG pseudopotentials to find an optimal set of weights and biases w, that is:

(6) $$ℒ\left({\tilde{z}}_{\text{leads }}\left(t\right),{\tilde{z}}_{\text{numerical }}\left(t\right);\hat{w}\right)=\underset{\hat{w}}{\text{argmin}}\left[∥{\tilde{z}}_{\text{leads }}\left(t\right)-{\tilde{z}}_{\text{numerical }}{\left(t\right)∥}_{{\text{L}}^{2}\left(0,T\right)}^{2}\right],$$

where ${\tilde{z}}_{\text{leads}}(t)\in [-1,1{]}^{9}$ represents BLNM outputs and ${\tilde{z}}_{\text{numerical}}(t)\in [-1,1{]}^{9}$ defines the physicsbased numerical simulations, both in non-dimensional form. Time $\tilde{t}\in [0,\;1]$ and model parameters ${\tilde{\theta }}_{EP}\in [-1,1{]}^{N_{𝒫}}$ are also normalized during the training and testing phases. We refer to [53] for a detailed description of all the properties related to BLNMs that enable them to effectively learn complex physical processes.

### 3.4 Parameter estimation

We employ our trained BLNM to find a set of model parameters ${\tilde{\theta }}_{EP}$ that matches $z_{ECG}(t)\in R^{12}$ with $z_{\text{clinical}}(t)\in R^{12}$. Here, $z_{ECG}(t)$ is the vector of BLNM physical outputs $z_{\text{leads}}(t)$ manipulated according to Equations (3) and (4) to generate the full 12-lead ECGs, and $z_{\text{clinical}}(t)$ is the clinically measured ECG data vector. In particular, we perform derivative-free optimization by employing the Nelder-Mead method [33], where we specify a loss function given by the MSE of the mismatch between the trained surrogate vs. clinical ECG potentials, that is:

(7) $$ℒ\left(z_{\text{ECG}}\left(t\right),z_{\text{clinical }}\left(t\right)\right)=∥z_{\text{ECG}}\left(t\right)-z_{\text{clinical }}{\left(t\right)∥}_{{\text{L}}^{2}\left(0,T\right)}^{2},$$

which leads to a set of calibrated model parameters ${\tilde{\theta }}_{EP}^{NM}$ and corresponding 12-lead ECGs $z_{ECG}^{NM}(t)$. We initialize our optimization algorithm with a random set of model parameters ${\tilde{\theta }}_{\text{init}}\in [-1,1{]}^{N_{𝒫}}$. We repeat the optimization process 100 times and we average the model parameters obtained during the different trials in order to get ${\tilde{\theta }}_{EP}^{NM}$.

### 3.5 Sensitivity analysis

We perform a variance-based sensitivity analysis using Shapley effects [56] in order to quantify the importance of each model parameter in fitting patient-specific 12-lead ECGs during the inference process.

Specifically, we employ Sklar’s theorem [12] to define the input multivariate distribution, which is given by a Gaussian copula and a series of $N_{𝒫}$ marginals defined by standard normal distributions centered in ${\tilde{\theta }}_{EP}^{NM}$, that is $𝒩\left({\tilde{\theta }}_{EP}^{NM,i},\;0.2\right)$ for $i=1,\ldots ,N_{𝒫}$.

Due to the high computational costs associated with testing all the different combinations of the features, we consider the random (rather than the exact) version of the algorithm to compute Shapley values. We monitor the expected marginal contribution of each model parameter to the BLNM prediction with respect to observations, that is the MSE of Equation (7). We fix 2000 permutations, 500 bootstrapped samples, and 50 samples to estimate conditional variance for 3 times.

### 3.6 Uncertainty quantification

We employ a BLNM within HMC [5] to calibrate model parameters and to perform inverse uncertainty by matching observed 12-lead ECGs from patient-specific recordings. HMC is a Markov Chain Monte Carlo (MCMC) method that aims at finding an approximation of the posterior distribution $P\left({\tilde{\theta }}_{EP}|x\right)$, given a certain prior probability distribution $P\left({\tilde{\theta }}_{EP}\right)$ with respect to the model parameters in non-dimensional form ${\tilde{\theta }}_{EP}\in [-1,1{]}^{N_{𝒫}}$. Specifically, we employ the No-U-Turn Sampler (NUTS) extension of HMC, which automatically adapts the number of steps to estimate the posterior distribution [21]. This algorithm, which shares and enhances some of the features of sequential [26] and differential evolution [61] MCMC, works well with high-dimensional target distributions, possibly presenting correlated dimensions. Moreover, HMC reaches convergence using a reduced amount of samples with respect to vanilla MCMC [21]. For further details about the mathematical derivation of HMC and its application to cardiac simulations we refer to [54].

We run 4 chains with 1,000 adaptation samples in the warm-up phase and 1,000 effective samples to estimate the posterior distribution, with a fixed 90% acceptance rate. For all model parameters, we consider prior distributions

(8) $$\mathbb{P}\left({\tilde{\theta }}_{\text{EP}}^{i}\right)\sim U\left({\tilde{\theta }}_{\text{EP}}^{\text{NM},\text{i}}-\iota ,{\tilde{\theta }}_{\text{EP}}^{\text{NM},\text{i}}+\iota \right)\text{ for }i=1,\ldots ,N_{𝒫},$$

where ${\tilde{\theta }}_{EP}^{NM}\in [-1,1{]}^{N_{𝒫}}$ is the initial guess obtained with the Nelder-Mead method. We always make sure that model parameters reside within the [−1, 1] range. We set ι=0.2. Even though NUTS allows for many different initialization protocols, such as maximum a posteriori (MAP) or maximum likelihood estimation (MLE), we consider an initial random seed for each chain. This is motivated by the sensitivity of MAP and MLE over multiple runs, especially when several model parameters are calibrated with respect to noisy or highly varying time-dependent QoIs, which is the case for ECG recordings.

Several sources of uncertainty can be considered. These include model uncertainties (e.g., the discrepancy between the actual physical phenomenon and the high-fidelity model), the discretization error introduced when solving the differential equations, the surrogate modeling error of reduced-order models, and the measurement errors that intrinsically affect clinical ECG recordings (i.e., the sensitivity of the instrument used during the clinical test, variations in lead placement position by clinicians, and patient-specific factors such as breathing and motion). However, in our inverse uncertainty quantification process, we only include the measurement error and the approximation error introduced by the transition from the high-fidelity model to the BLNM-based surrogate model. In particular, we consider a multivariate normal distribution centered in the BLNM predictions $z_{ECG}(t)$ for the given patient-specific observations $z_{clinical}(t)$, which reads:

(9) $$z_{\text{clinical }}\left(t\right)\sim 𝒩\left(z_{\text{ECG}}\left(t\right),\sigma_{\text{meas }}^{2}I+k\left(\tilde{t},{\tilde{t}}^{\prime };\sigma_{\text{GP}},l_{\text{GP}}\right)\right).$$

$\sigma_{\text{meas}}=0.1$ is the a priori fixed standard deviation dictating the measurement error [15, 69], whereas $k\left(\tilde{t},\tilde{t};\sigma_{GP},l_{GP}\right)=\sigma_{GP}^{2}\;exp\left(\frac{-{∥\tilde{t}-{\tilde{t}}^{2}∥}^{2}}{2l_{GP}^{2}}\right)$ is the exponentiated quadratic kernel of a zero-mean Gaussian process $𝒢𝒫\left(0,\;k\left(\tilde{t},\tilde{t};\sigma_{GP},l_{GP}\right)\right)$ [46]. Amplitude $\sigma_{GP}\sim 𝒩(0.01,\;1.0)$ and correlation length $l_{GP}\sim 𝒩(0.01,\;1.0)$ are additional hyperpriors tuned during HMC to quantify the surrogate modeling error, which may change according to the specific observation. Vectors $\tilde{t}$ and $\tilde{t}$ represent discrete time points in the [0, 1] interval.

A full covariance matrix in the multivariate normal distribution allows us to model the correlation among different leads. We evaluate convergence of the HMC chains by checking that the Gelman-Rubin diagnostic provides a value less than 1.1 for all the model parameters ${\tilde{\theta }}_{EP},l_{GP}$ and $\sigma_{GP}$ [6, 17, 65].

### 3.7 Software and hardware

All electrophysiology simulations are performed at the Stanford Research Computing Center using svFSIplus [70], a C++ high-performance computing multiphysics and multiscale finite element solver for cardiac and cardiovascular modeling. This solver is part of the SimVascular software suite for patient-specific cardiovascular modeling [64].

We train the NNs by using BLNM.jl [22, 45], Julia library for scientific machine learning which is publicly available under MIT License at https://github.com/StanfordCBCL/BLNM.jl. This library leverages Hyperopt.jl [3] for parallel hyperparameter optimization by combining the Message Passing Interface (MPI) with Open Multi-Processing (OpenMP) on physical and virtual cores, respectively.

We perform sensitivity analysis and parameter estimation with uncertainty quantification using GlobalSensitivity.jl [11] and Turing.jl [16], respectively, which both exploit OpenMP and vectorized operations to speed-up computations. The code for sensitivity analysis and Bayesian parameter estimation is available within BLNM.jl as a test case.

Furthermore, this public repository contains the dataset encompassing all the electrophysiology simulations used for the training and testing phases, along with the patient-specific 12-lead ECGs.

## 4 Discussion

We present a complete computational pipeline to build digital twins of cardiac electrophysiology for congenital heart disease in pediatrics. This cohort of patients is understudied in cardiology [32, 62], as multiphysics and multiscale numerical simulations are mostly focused on adults with certain sets of pathologies, such as dilated, ischemic and hypertrophic cardiomyopathy, arrhythmias or bundle branch block [36, 38, 52, 58].

In this pipeline, we leverage biophysically detailed and anatomically accurate computational electrophysiology models, a recently proposed scientific machine learning tool for surrogate modeling, and robust Bayesian inference methods for personalized calibration of model parameters to match clinical 12-lead ECGs of an HLHS pediatric patient. We certify the impact and reliability of our estimation against clinical recordings by integrating fast and effective sensitivity analysis and uncertainty quantification. We run electrophysiology simulations with the estimated model parameters in order to investigate different scenarios of clinical interest in silico. We conclude that this pediatric patient presents activation and repolarization patterns similar to a left bundle branch block, where the interventricular dyssynchrony and the geometrical personalization of the Purkinje network play a minor role with respect to conductances and conductivities, even for QRS complex calibration.

Image processing allows us to get all the anatomy-specific features of this pediatric patient and our calibration of cell-to-organ level model parameters enables patient-specific electrophysiology simulations. Nevertheless, given the non-convexity of the optimization problem, it is important to stress that the final set of model parameters might not be unique and there could be other choices that lead to similar approximation errors against clinical recordings. Indeed, we notice that changing random seeds or trying different optimizers, such as second-order local BFGS or even global Adaptive Differential Evolution [68], may have an influence on the initial parameter estimation. These options are available within the BLNM.jl library. However, these effects are accounted for and mitigated by averaging many different trials and by running uncertainty quantification.

Performing ad-hoc sensitivity analysis for a specific parameter calibration provides individualized information, as these assessments may change on a patient to patient basis. Furthermore, we underline that sensitivity and practical identifiability (or trustworthiness) of model parameters are generally correlated. For instance, the maximum rapid delayed rectifier current conductance $G_{Kr}$ and the level of interventricular dyssynchrony $t_{LV}^{stim}$ have the lowest relative impact on this 12-lead ECGs personalization (see Figure 4) and present the highest degree of uncertainty, that is a wider posterior distribution, among all physics-based model parameters (see Figure 5).

While the computational pipeline encompasses several rigorous steps, the physics-based model still requires high-performance computing and longer computational times compared to other approaches for digital twinning on adults [19, 20], which rely on more phenomenologically-based models but do not include robust methods for sensitivity analysis and uncertainty quantification. However, the monodomain equation, coupled with the ten Tusscher-Panfilov ionic model, provides an accurate mathematical model, where relevant model parameters with a direct physiological interpretation can be properly tuned. Moreover, its higher computational costs could be mitigated in future work by novel numerical methods in the framework of matrix-free [1] and Isogeometric Analysis [7].

A limitation of the presented approach lies in the lack of experimental validation of the parameter calibration process. Indeed, mathematical modeling of congenital heart disease requires several assumptions due to the current lack of information in pediatric populations regarding fiber orientation, Purkinje structure, ionic current conductances, and conduction velocities. Future studies should incorporate these data as they become available. Nevertheless, our estimations are robust, account for uncertainty quantification and are widely contained within the range explored by the electrophysiology simulations (see Table 1 and 3, along with Figure 5).

In future developments, we aim to encode anatomical variability and different CHDs, such as Tetralogy of Fallot, transposition of great arteries or atrial and ventricular septal defects within BLNMs. In this manner, the computationally expensive offline phase dictated by accurate numerical simulations and the training of the NN can be performed only once before being applied to new patients. Robust parameter estimation and uncertainty quantification will be then feasible for those CHDs within minutes, compatible with the time frame required by the clinical practice.

## Figures and Tables

**Table 1:** Parameter space for cardiac electrophysiology sampled via latin hypercube for the numerical simulations performed with the physics-based mathematical model.

Parameter	Description	Range	Units
*G*_CaL_	Maximal Ca^2^+ current conductance	[1.99e-5, 7.96e-5]	cm ms^−1^ *μ*F^−1^
*G*_NA_	Maximal Na+ current conductance	[7.42, 29.68]	nS pF^−1^
*G*_Kr_	Maximal rapid delayed rectifier current conductance	[0.08, 0.31]	nS pF^−1^
*D*_ani_	Anisotropic conductivity	[0.008298, 0.033192]	mm^2^ ms^−1^
*D*_iso_	Isotropic conductivity	[0.002766, 0.011064]	mm^2^ ms^−1^
*D*_purk_	Purkinje conductivity	[1.0, 3.5]	mm^2^ ms^−1^
$t_{LV}^{\text{stim }}$	Purkinje left bundle stimulation time	[0, 100]	ms

**Table 2:** Branched Latent Neural Map hyperparameter tuning. Original hyperparameter ranges and optimized hyperparameter values for the final training stage.

BLNM	Hyperparameters				Trainable parameters
	layers	neurons	number of states	disentanglement level	# parameters
tuning	{1 … 8}	{10 … 30}	{9 … 12}	{1 … N_layers_}	
final	7	19	10	2	2,398

**Table 3:** Parameter estimation. Calibration with the optimized BLNM for cell-to-organ level model parameters of the physics-based mathematical model. The MSE between the BLNMs predictions and the clinical recordings, in non-dimensional form, is 0.16.

Parameter	Value	Units
*G*_CaL_	2.94e-5	cm ms^−1^ *μ*F^−1^
*G*_NA_	15.58	nS pF^−1^
*G*_Kr_	0.15	nS pF^−1^
*D*_ani_	0.03	mm^2^ ms^−1^
*D*_iso_	0.01	mm^2^ ms^−1^
*D*_purk_	1.96	mm^2^ ms^−1^
$t_{LV}^{\text{stim }}$	43.3	ms

**Table 4:** Computational resources. Summary of the computational times and resources to generate the electrophysiology simulations with the physics-based model, to train the BLNM, to compute Shapley values for sensitivity analysis and to perform Bayesian parameter estimation with uncertainty quantification on 12-lead ECGs.

Task	Computational resources	Execution time
Segmentation and mesh generation (one patient)	1 core	10 minutes
200 electrophysiology simulations	336 cores	1 day
BLNM hyperparameter tuning (50 confs, 10,000 iters)	5 cores	20 hours
BLNM final training (50,000 iters)	1 core	2 hours and 30 minutes
Parameter estimation (100 trials)	1 core	2 minutes
Sensitivity analysis (Shapley values)	1 core	20 minutes
Uncertainty quantification (HMC, 4 chains)	4 threads	5 minutes
*Total*	-	*2 days*
